# Dietary supplementation with gamma-linolenic, linoleic and oleic acids decreases PPAR-gamma expression and helps the tetracycline derivative to reduce NOD2 expression in patients with acne vulgaris^☆^

**DOI:** 10.1016/j.abd.2020.11.016

**Published:** 2022-01-17

**Authors:** Clarissa Prati, Emily Ferreira Salles Pilar, Andre Cartel, João Bayma Galvão Pitoni, Cidia Vasconcellos, Adilson da Costa

**Affiliations:** a Instituto de Assistência Médica ao Servidor Público Estadual, São Paulo, SP, Brazil; b Hospital de Clínicas, Universidade Federal do Rio Grande do Sul, Porto Alegre, RS, Brazil; c International Pediatric Clinics, Atlanta, GA, USA

Dear Editor,

Acne vulgaris (AV) is an immunoinflammatory dermatosis of the sebaceous glands (SG), inter-adnexal epithelium (IE) and hair follicles (HF); it shows lipid synthesis and cytokine expression by microorganism-cell interaction. In AV, toll-like receptors type 2 (TLR2) and 4 (TLR4), nucleotide oligomerization domain-like, type 1 (NOD1) and 2 (NOD2) and peroxisome proliferator-activated receptor gamma (PPARG) regulate the SG expression, cell differentiation and macronutrient metabolism.^1^ Dietary fatty acids (FAs) seem to influence AV through the imbalance of omega-3 and -6 FAs.^2^ Our hypothesis is that supplementation with gamma linolenic (GLA), linoleic (AL) and oleic (OA) acids, with or without lymecycline (LM), interferes with the expression of TLR1, TLR2, NOD1, NOD2, and PPARG in AV.

The present research was an open, prospective, randomized, non-placebo-controlled study, approved by the Research Ethics Committee of Pontifícia Universidade Católica de Campinas (PUC-Campinas; protocol n.387/09) and Faculdade de Medicina da Universidade de São Paulo (FMUSP; 229/10), at the Dermatology Service at PUC-Campinas; FMUSP Department of Dermatology; Experimental Pathology Laboratory of Universidade Federal do Rio Grande do Sul (UFRGS); and the Postgraduate Program in Health Sciences at Instituto de Assistência Médica ao Servidor Público Estadual (IAMSPE) in São Paulo, resulting in the Doctorate theses of the first and last authors. Financial support was provided by Fundo de Apoio da Sociedade Brasileira de Dermatologia (Funaderm) and a donation of LM (Tetralysal®, 300 mg) and GLA/LA/OA (Tiliv L, 1000 mg; 480 mg/day, 1200 mg/day and 510 mg/day, respectively, of GLA/LA/OA) by Galderma Brasil Ltda., São Paulo, SP, Brazil, and Arese Pharma Ltda., Valinhos, SP, Brazil, respectively.

Forty-five male patients with papulopustular or cystic acne, aged between 13 and 38 years, read, understood, signed the free and Informed Consent Form and were randomized into three therapeutic groups (1:1:1) for 90 days: Group 1, LM 300 mg/day; Group 2, GLA/LA/OA 1000 mg/day; or Group 3, Group 1+Group 2. Papules, pustules and cysts were counted at Visits 1, Day 0 (D0); 2, D30; 3, D60; and 4, D90. A biopsy of the papulopustular lesion was performed on D0 and D90 for immunohistochemical analysis of TLR1, TLR2, NOD1, NOD2, and PPARG, evaluated by three pathologists using the following grading score: non-existent (0), weak (1), medium (2) and strong (3).

The means obtained before and after treatment were compared between groups and lesions. Quantitative variables were described by mean and standard deviation, comparing those with a symmetrical distribution by analysis of variance (ANOVA), followed by Tukey’s posthoc; intergroup evaluation was carried out with the Kruskal-Wallis test for non-parametric data; and intragroup evaluation with Wilcoxon test. Descriptive statistics were used, with standard deviation, minimum, maximum ​​, and median values, with a significance level of 5% (p < 0.05) using the statistical package IBM SPSS version 20.

Of the 45 subjects, 36 (80%) completed the study: 11 from Group 1; 13 from Group 2; and 12 from Group 3. There was no statistical difference between the groups (p = 0.626). The ratio of subjects with papulopustular and cystic acne was similar for Groups 1, 2 and 3, and there were no differences regarding phototypes (p = 0.548) and time of disease evolution (p = 0.959; Table 1).

**Table 1:** Characteristics of subjects with AV included in the study.

Variables	Groups of treatment			p-value
	Group 1 (n = 11)	Group 2 (n = 13)	Group 3 (n = 12)	
Age (years)				0.626
Mean(SD)	17.5 (3.9)	17.7 (4.7)	19.6 (6.2)	
Median (Min–Max)	17 (13 – 28)	16.5 (16 – 31)	17 (15 – 38)	
AV degree				0.327
Papulopustular	9 (81.8)	11 (84.6)	10 (83.3)	
Cysts	2 (18.2)	2 (15.4)	2 (16.7)	
Phototype				0.548
II	3 (27.2)	3 (23.1)	3 (25)	
III	4 (36.4)	2 (15.4)	3 (25)	
IV	4 (36.4)	8 (61.5)	6 (50.0)	
Time of disease (years)				0.959
Mean (SD)	5.3 (2.8)	5.6 (3.6)	6.4 (4.4)	
Median (Min–Max)	4.5 (2 – 12)	4.5 (2 – 15)	5.0 (3 – 18)	

SD, Standard Deviation; Min, Minimum; Max, Maximum; n, Number; p, Probability of observing a result as extreme or more extreme than that of the sample, assuming that the null hypothesis is true; AV, Acne Vulgaris.

A significant decrease was observed in the number of comedones and cysts throughout the study (p < 0.001), with no difference between the groups and no alterations in comedones and cysts. The pustules decreased significantly (p < 0.001), with no effect of time, difference between the groups (p = 0.049; Table 2). However, the papules and total AV lesions varied between the study groups (p = 0.049 and p = 0.011; group*Visit interaction; Table 2). There was a decrease in comedones and pustules in all groups and visits when compared to D0, except between D60 and D90 (p = 0.966). For the cysts, there was a decrease, except from D30 on (Table 3).

**Table 2:** Descriptive statistics of lesions according to group and time of treatment.

Visit	Number of lesions	Group 1	Group 2	Group 3	p-value
D0	Comedones				0.328
	Mean (SD)	18.4 (5.7)	23.2 (10.7)	25.5 (13.0)	
	Median (Min–Max)	18.5 (12 – 29)	23.0 (5 – 38)	27.0 (5 – 46)	
	Papules				0.162
	Mean (SD)	11.3 (4.9)	13.6 (4.6)	14.3 (7.3)	
	Median (Min–Max)	9.5 (6 – 22)	12.0 (8 – 25)	12.0 (6 – 35)	
	Pustules				0.290
	Mean (SD)	7.3 (4.9)	10.1 (5.4)	9.4 (6.5)	
	Median (Min–Max)	8 (2 – 18)	9 (3 – 22)	8 (2 – 26)	
	Cysts				0.496
	Mean (SD)	1.0 (2.0)	0.4 (0.9)	2.9 (5.5)	
	Median (Min–Max)	0 (0 – 6)	0 (0 – 3)	0 (0 – 16)	
	Any lesion				0.156
	Mean (SD)	38.0 (14.5)	47.2 (16.4)	52.1 (23.0)	
	Median (Min–Max)	38 (20 – 71)	41.5 (24 – 83)	49 (18 – 99)	
D30	Comedones				0.175
	Mean (SD)	14.8 (4.6)	18.9 (8.7)	20.3 (9.1)	
	Median (Min–Max)	14 (8 – 22)	18 (4 – 32)	22 (3 – 33)	
	Papules				0.156
	Mean (SD)	7.3 (4.2)	9.5 (3.4)	8.1 (5.3)	
	Median (Min–Max)	7 (2 – 16)	9 (4 – 16)	7 (3 – 24)	
	Pustules				0.374
	Mean (SD)	3.8 (3.5)	5.6 (3.0)	5.4 (4.4)	
	Median (Min–Max)	3 (0 – 10)	5.5 (1 – 11)	4 (0 – 15)	
	Cysts				0.160
	Mean (SD)	0.3 (0.9)	0.0 (0.0)	1.5 (3.5)	
	Median (Min–Max)	0 (0 – 3)	0 (0 – 0)	0 (0 – 12)	
	Any lesion				0.142
	Mean (SD)	26.1 (10.3)	33.9 (14.0)	35.4 (15.3)	
	Median (Min–Max)	26.5 (10 – 49)	31.5 (12 – 57)	36 (12 – 67)	
D60	Comedones				0.296
	Mean (SD)	12.8 (4.0)	17.5 (9.0)	15.6 (6.6)	
	Median (Min–Max)	13 (8 – 20)	16 (4 – 33)	16 (3 – 26)	
	Papules				0.009
	Mean (SD)	5.1 (2.5)	8.9 (3.7)	5.1 (3.9)	
	Median (Min–Max)	5 (1 – 10)	8.5 (3 – 15)	5 (0 – 16)	
	Pustules				0.022
	Mean (SD)	2.6 (1.9)	5.6 (3.2)	2.4 (2.9)	
	Median (Min–Max)	3.5 (0 – 5)	5 (1 – 12)	1 (0 – 8)	
	Cysts				0.149
	Mean (SD)	0.0 (0.0)	0.0 (0.0)	0.6 (1.7)	
	Median (Min–Max)	0 (0 – 0)	0 (0 – 0)	0 (0 – 6)	
	Any lesion				0.060
	Mean (SD)	20.4 (6.7)	31.9 (12.9)	23.8 (10.6)	
	Median (Min–Max)	22.5 (9 – 27)	32.5 (13 – 55)	25.5 (7 – 43)	
D90	Comedones				0.19
	Mean (SD)	11.8 (3.8)	16.7 (8.0)	13.4 (6.3)	
	Median (Min–Max)	12 (6 – 20)	14 (5 – 30)	12 (3 – 25)	
	Papules				0.003
	Mean (SD)	4.5 (2.7)	8.4 (4.2)	3.8 (2.3)	
	Median (Min–Max)	3.5 (1 – 10)	9 (3 – 18)	3 (0 – 8)	
	Pustules				0.003
	Mean (SD)	2.2 (2.6)	6.2 (4.1)	1.6 (1.7)	
	Median (Min–Max)	1.5 (0 – 9)	7 (1 – 13)	1 (0 – 4)	
	Cysts				<0.001
	Mean (SD)	0.0 (0.0)	0.0 (0.0)	0.6 (1.7)	
	Median (Min–Max)	0 (0 – 0)	0 (0 – 0)	0 (0 – 6)	
	Any lesion				0.006
	Mean (SD)	18.4 (6.5)	31.4 (11.6)	18.8 (9.0)	
	Median (Min–Max)	18.5 (8 – 27)	36 (11 – 48)	18.5 (6 – 32)	

SD, Standard Deviation; Min, Minimum; Max, Maximum; n, Number; p, Probability of observing a result as extreme or more extreme than that of the sample, assuming that the null hypothesis is true.

**Table 3:** Multiple comparisons of numbers of lesions at the visits for the treatment groups.

Lesion	Comparison	Estimated mean difference	Standard Error	p-value
Comedones	D0 *vs.* D30	4.450	0.674	<0.001
	D0 *vs.* D60	7.175	1.095	<0.001
	D0 *vs.* D90	8.500	1.232	<0.001
	D30 *vs.* D60	2.725	0.556	<0.001
	D30 *vs.* D90	4.050	0.748	<0.001
	D60 *vs.* D90	1.325	0.368	0.005
Pustules	D0 *vs.* D30	4.000	0.473	<0.001
	D0 *vs.* D60	5.425	0.669	<0.001
	D0 *vs.* D90	5.625	0.916	<0.001
	D30 *vs.* D60	1.425	0.434	0.012
	D30 *vs.* D90	1.625	0.702	0.113
	D60 *vs.* D90	0.200	0.429	0.966
Cysts	D0 *vs.* D30	0.850	0.224	0.001
	D0 *vs.* D60	1.225	0.301	<0.001
	D0 *vs.* D90	1.450	0.352	<0.001
	D30 *vs.* D60	0.375	0.224	0.341
	D30 *vs.* D90	0.600	0.301	0.197
	D60 *vs.* D90	0.225	0.224	0.747

D30, 30 days of study; D60, 60 days of study; D90, 90 days of study; p, Probability of observing a result as extreme or more extreme than that of the sample, assuming that the null hypothesis is true.

On immunohistochemistry, there was no difference between the groups and analyzed histopathological sites on D0. On D90, there was a NOD2 difference in the IE between the Groups (Group 1 with higher values ​ than Groups 2 and 3). The median of NOD2 in the IE was two in the three groups, with a maximum value in Group 1 surpassing the others, indicating a somewhat higher distribution of values ​​in Group 1. In the intragroup comparisons, there was a decrease in PPARG in SG in Group 2 (p = 0.016) and a significant increase in NOD2 in HF in Group 1 (p = 0.011; Table 4).

**Table 4:** Comparative table between and within groups for the assessment by the score.

	Group 1 (n = 11)			Group 2 (n = 13)			Group 3 (n = 12)				
	D0	D90	P^c^	D0	D90	P^c^	D0	D90	P^c^	PD0^d^	PD90^d^
Epithelium											
PPARG	2 (0–3)	2 (0–3)	0.603	2 (0–3)	1 (0–3)	0.088	3 (0–3)	1 (0–3)	0.184	0.340	0.482
NOD1	3 (2–3)	3 (2–3)	0.317	3 (2–3)	3 (1–3)	0.480	3 (1–3)	3 (1–3)	0.180	0.797	0.177
NOD2	2 (1–3)	2 (2–3)^a^	0.063	2 (0–3)	2 (1–2)^b^	0.102	1.5 (0–3)	2 (1–2)^b^	0.999	0.500	0.001^d^
TRL2	2 (0–3)	2 (1–3)	0.317	1 (1–3)	2 (0–3)	0.480	1 (1–3)	1 (0–3)	0.739	0.370	0.794
TRL4	1 (0–3)	1 (0–2)	0.999	1 (0–3)	1 (0–3)	0.627	1 (0–3)	1 (0–3)	0.720	0.404	0.503
Gland											
PPARG	2 (1–3)	2 (1–3)	0.180	2 (2–3)	2 (0–3)	0.016^c^	2 (1–3)	2 (1–3)	0.132	0.146	0.091
NOD1	2 (1–3)	2 (1–3)	0.366	2 (1–3)	2 (0–3)	0.589	2 (1–3)	2 (2–3)	0.096	0.696	0.197
NOD2	2 (0–3)	2 (1–3)	0.107	2 (0–3)	2 (1–3)	0.317	1 (1–2)	2 (1–3)	0.107	0.331	0.560
TRL2	1 (0–3)	1 (0–2)	0.084	0 (0–3)	1 (0–2)	0.480	1 (0–2)	1 (0–3)	0.271	0.083	0.777
TRL4	0 (0–2)	0 (0–2)	0.999	0 (0–2)	0 (0–2)	0.890	0 (0–3)	1 (0–3)	0.546	0.440	0.067
Follicle											
PPARG	0 (0–3)	1 (0–2)	0.739	1 (0–3)	0 (0–3)	0.429	1 (0–2)	1 (0–3)	0.454	0.941	0.299
NOD1	3 (1–3)	3 (1–3)	0.705	2 (0–3)	2 (0–3)	0.874	2 (0–3)	2 (1–3)	0.386	0.405	0.499
NOD2	1 (0–2)	2 (0–3)	0.011^c^	1 (0–2)	1 (0–2)	0.705	0.5 (0–1)	1 (0–2)	0.084	0.082	0.092
TRL2	1 (0–3)	1 (0–2)	0.236	0 (0–3)	1 (0–2)	0.999	1 (0–1)	1 (0–2)	0.206	0.388	0.449
TRL4	0 (0–2)	1 (0–2)	0.480	0 (0–2)	0 (0–3)	0.726	0.5 (0–3)	1 (0–2)	0.850	0.565	0.304

Data presented as median (minimum–maximum).

^a,b^Different letters represent statistically different distributions.

^c^Wilcoxon test.

^d^Kruskal Wallis test.

The reduction of inflammatory lesions in the Groups with LM undergoing treatment (Groups 1 and 3) was predicted, as it is one of the tetracyclines of choice in the management of inflammatory AV, with high skin penetration.^3^, ^4^

Costa et al., in 2007, pointed out a clinical non-response with the use of GLA/LA/OA supplementation, compared to placebo, in AV, but a possible improvement in the SG size, seen in pre-and post-treatment biopsies.^5^ The present study, however, indicated a possible usefulness of GLA/LA/OA for comedones and cysts, confirming Rustin's remote assumptions.^6^

NODs are activated by bacterial muramyl peptidoglycans, combined or not with TLRs. Thus, the result observed in NOD2 may have come from the control of microorganism-mediated inflammation, as the overexpression of NOD2 reduces clonal proliferation.^7^, ^8^

PPARG acts on sebocyte modulation. Its GLA/LA/OA-induced SG reduction may be the cause of the increase in lauric, myristic, and palmitic acids under equal therapy, suggesting the same role in NOD2-lowering effects in the IE.^9^

GLA/LA/OA were used In Groups 2 and 3. Therefore, we suggest that their administration causes PPARG reduction in sebocytes in Group 2 and NOD2 in the IE of both Groups 2 and 3, reinforcing the hypothesis of beneficial supplementation with GLA/LA/OA for comedonal and cystic AV.^5^, ^6^, ^7^ Therefore, FAs with great bactericidal potential could reduce the concentrations of *Cutibacterium acnes* and, thus, the expression of bacterium-activated receptors. Interestingly, isolated LM increased NOD2 labeling in HF, requiring further elucidative studies. As PPARG and NOD2 are inversely related to papules in Group 3, perhaps they can be recruited in the early stages of the dermatosis.

Therefore, it is concluded that daily administration of LM and/or GLA/LA/OA interferes with the pro-inflammatory markers in AV, and supplementation with GLA/LA/OA could be an adjuvant in the treatment of AV.

## Financial support

This publication was made possible with the support from *Fundo de Apoio à Dermatologia (Funaderm) da Sociedade Brasileira de Dermatologia*, Galderma Brasil Ltda., São Paulo, SP, Brazil, and Arese Pharma Ltda., Valinhos, SP, Brazil.

## Authors’ contributions

Clarissa Prati: Statistical analysis; approval of the final version of the manuscript; design and planning of the study; drafting and editing of the manuscript; collection, analysis, and interpretation of data; critical review of the literature.

Emily Ferreira Salles Pilar: Approval of the final version of the manuscript; collection, analysis, and interpretation of data; effective participation in research orientation.

Andre Cartel: Approval of the final version of the manuscript; collection, analysis, and interpretation of.

João Bayma Galvão Pitoni: Approval of the final version of the manuscript; collection, analysis, and interpretation of data.

Cidia Vasconcellos: Approval of the final version of the manuscript; effective participation in research orientation; critical review of the manuscript.

Adilson da Costa: Approval of the final version of the manuscript; design and planning of the study; drafting and editing of the manuscript; effective participation in research orientation; critical review of the manuscript.

## Conflicts of interest

None declared.
